# Mass‐Manufactured Gradient Plasmonic Metasurfaces for Enhanced Mid‐IR Spectrochemical Analysis of Complex Biofluids

**DOI:** 10.1002/adma.202504355

**Published:** 2025-08-29

**Authors:** Samir Rosas, Shovasis Kumar Biswas, Wihan Adi, Furkan Kuruoglu, Aidana Beisenova, Manish S. Patankar, Filiz Yesilkoy

**Affiliations:** ^1^ Department of Biomedical Engineering University of Wisconsin–Madison Madison WI 53706 USA; ^2^ Department of Electrical and Computer Engineering University of Wisconsin‐Madison Madison WI 53706 USA; ^3^ Department of Physics Faculty of Science Istanbul University Vezneciler Istanbul 34134 Turkey; ^4^ Department of Obstetrics and Gynecology University of Wisconsin–Madison Madison WI 53792 USA

**Keywords:** biosensors, mid‐infrared spectroscopy, plasmonic metasurfaces, surface‐enhanced infrared absorption spectroscopy

## Abstract

Mid‐infrared (Mid‐IR) spectroscopy offers powerful label‐free molecular analysis capabilities but faces significant challenges when analyzing complex biological samples. Here, a transformative surface‐enhanced infrared absorption spectroscopy (SEIRAS) platform is presented that overcomes fundamental limitations through key innovations. First, high‐throughput wafer‐scale fabrication of mid‐IR plasmonic micro‐hole‐array (MHA) metasurfaces is demonstrated on free‐standing silicon nitride (Si_3_N_4_) membranes, yielding ≈400 sensor chips per 6‐inch wafer. Second, the gradient MHA metasurface design supports spectrally cascaded plasmonic modes, generating over 400 sharp resonance peaks across the 1200–2000 cm^−1^ fingerprint region. This approach enables comprehensive molecular fingerprinting using simple imaging optics in transmission mode. Third, the SEIRAS platform is validated using a model polymer system and clinical peritoneal fluid samples from ovarian cancer patients, demonstrating its capability to resolve complex molecular signatures in real biological specimens. The platform's dense spectral coverage ensures optimal on‐resonance enhancement across the broad fingerprint region, revealing previously obscured vibrational bands that conventional IR spectroscopy cannot distinguish. By combining high‐throughput fabrication with simplified optical readout and the capability to analyze complex biological samples, this work establishes a foundation for translating SEIRAS technology into practical biomedical applications, promising a real‐world impact.

## Graphical abstract

This article presents a surface‐enhanced infrared absorption spectroscopy (SEIRAS) platform using mass‐manufactured gradient plasmonic metasurfaces, overcoming limitations in analyzing real‐world biosamples. The technology achieves 20‐fold signal enhancement and demonstrates superior molecular fingerprinting capabilities. By combining high‐throughput fabrication with simplified optical readout and capability to analyze human biofluids, this work establishes a foundation for translating SEIRAS technology into practical biomedical applications.

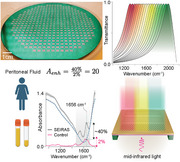

## 1 Introduction

Label‐free vibrational spectroscopy in the mid‐infrared (mid‐IR) spectrum (𝜆 = 2.5–25 µm) is a powerful tool for noninvasive analysis of biological samples without lengthy sample staining and preparation. This technique enables the detection of biomolecules based on their unique spectral fingerprints, where molecular vibrational energy states are inscribed as absorption peaks.^[^ ^1^, ^2^ ^]^ Spectrochemical fingerprinting is particularly promising for biomedical diagnostics,^[^ ^3^, ^4^ ^]^ disease monitoring,^[^ ^5^, ^6^ ^]^ and biomarker discovery^[^ ^7^, ^8^ ^]^ because it can simultaneously capture diverse molecular structures from biospecimens. However, when analyzing complex biological samples, conventional mid‐IR absorption spectroscopy (MIRAS) faces significant challenges. Specifically, heterogeneity in sample thickness and composition^[^ ^9^ ^]^ and optical scattering^[^ ^10^ ^]^ distort and congest spectral data, impeding reliable chemometric analyses.^[^ ^11^ ^]^ Therefore, innovative analytical platforms capable of capturing accurate mid‐IR spectral fingerprints from real‐world samples are urgently needed.

To overcome these limitations, surface‐enhanced infrared absorption spectroscopy (SEIRAS) has been developed, offering significantly higher sensitivity through enhanced light‐matter interactions in nanophotonic cavities.^[^ ^12^, ^13^ ^]^ To date, numerous SEIRAS technologies have been proposed using antennas,^[^ ^14^, ^15^ ^]^ antenna clusters,^[^ ^9^, ^16^ ^]^ and their 2D arrays forming metasurfaces from metallic,^[^ ^17^ ^]^ dielectric,^[^ ^18^, ^19^, ^20^ ^]^ and 2D materials.^[^ ^21^ ^]^ By leveraging strong near‐field light localization, SEIRAS has been applied to analyze biological cells,^[^ ^22^ ^]^ tissues,^[^ ^9^ ^]^ and functional biomolecules, such as proteins,^[^ ^23^ ^]^ nucleic acids,^[^ ^24^ ^]^ carbohydrates.^[^ ^25^ ^]^ However, current SEIRAS platforms face three major challenges that limit their impact and hinder their practical applications.

First, nanostructured SEIRAS substrates typically rely on expensive and low‐throughput electron‐beam lithography fabrication on IR‐transparent windows like CaF_2_^[^ ^20^, ^26^, ^27^, ^28^ ^]^ and MgF_2_.^[^ ^29^ ^]^ Although these materials provide excellent optical access to plasmonic resonances, they present significant fabrication challenges. They are expensive, mechanically brittle, and incompatible with high throughput UV lithography techniques. As a result, the scalable production of metasurfaces fabricated on IR‐transparent windows has not been possible, obstructing their real‐world impact. Second, SEIRAS signals are usually embedded in the far‐field resonance distortions, such as amplitude damping, frequency shifting, and mode broadening. Typical mid‐IR antennas generate resonance peaks in the reflection mode requiring complex readout platforms, which obstruct the development of cost‐effective, robust, and portable sensors. Third, when measuring chemically complex samples using SEIRAS, accurate retrieval of absorption spectra in the broad fingerprint region requires alignment of photonic resonances to individual vibrational bands. This alignment is essential because the electromagnetic near‐field enhancement reaches its maximum at the central wavelength of the resonance peak. Furthermore, achieving on‐resonance SEIRAS ensures realistic symmetric band retrieval, in contrast to Fano‐like asymmetric profiles that emerge when the photonic resonance is detuned from the vibrational band.^[^ ^30^ ^]^

Some of these SEIRAS challenges have been addressed by recent developments in the nanophotonics field. For example, to eliminate the reliance on IR‐transparent substrates, Si^[^ ^18^, ^19^ ^]^ and Al_2_O_3_ membranes^[^ ^31^ ^]^ were used to fabricate metasurfaces for SEIRAS applications. To enable simple SEIRAS sensor platforms operating in transmission mode, we recently demonstrated transmissive resonances supported by Si membrane^[^ ^18^ ^]^ metasurfaces. Moreover, others proposed plasmonic devices with transmission resonances for SEIRAS applications.^[^ ^32^, ^33^, ^34^ ^]^ To enable on‐resonance SEIRAS across the fingerprint spectrum, gradient metasurfaces were developed where continuously tuned metasurface resonances enabled precise alignment with multiple vibrational bands on the same chip.^[^ ^35^, ^36^ ^]^ While these previous efforts tried to address individual limitations of the SEIRAS systems, a single platform to overcome all the problems at once has not yet been reported. Additionally, previous platforms are often validated using pure model molecules, such as PMMA or protein and DNA species, rather than complex biological samples, leaving their real‐world utility unproven.

**Figure 1:**
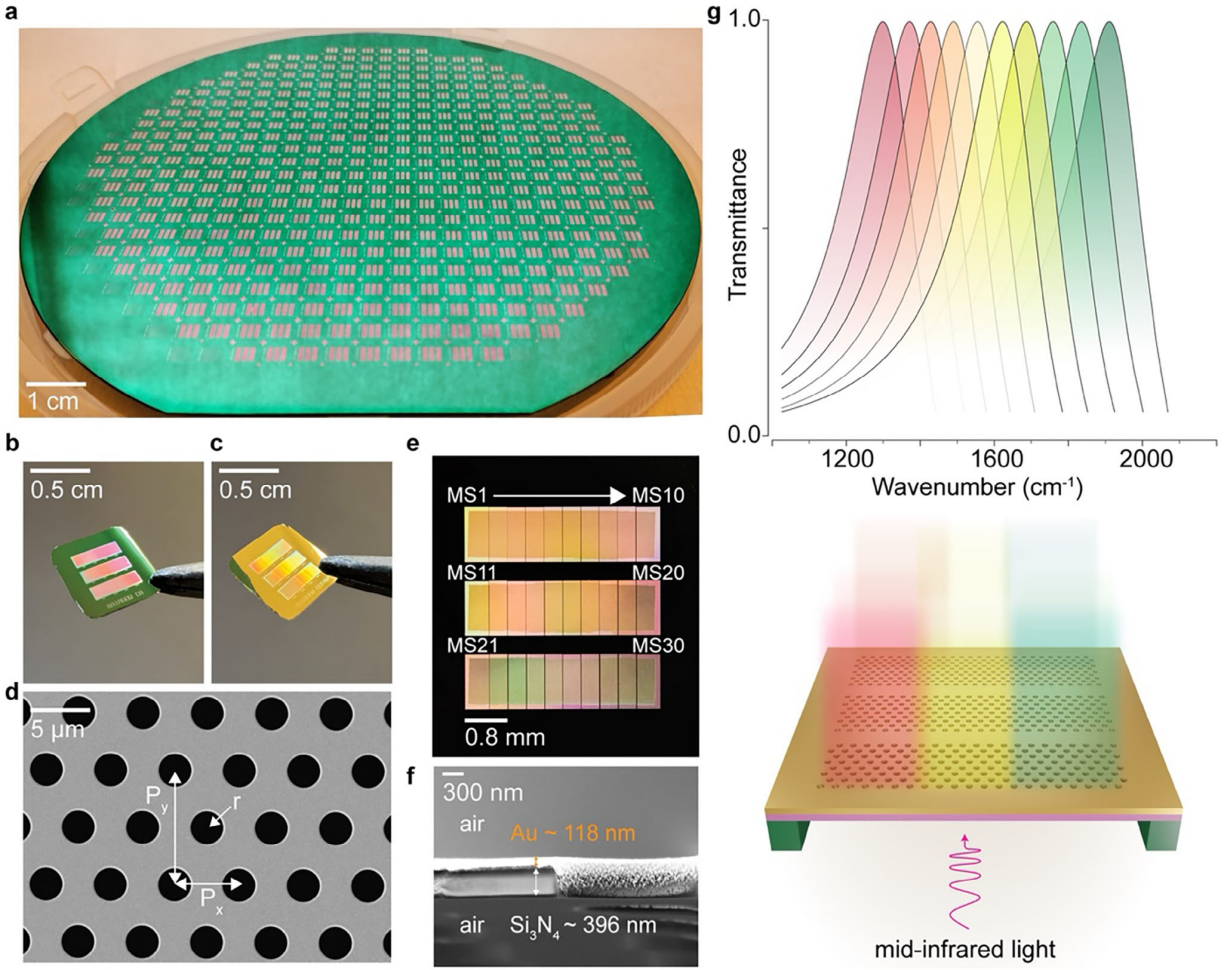
High‐throughput‐manufactured gradient micro‐hole array (MHA) metasurfaces. a) Photograph of a 6‐inch wafer carrying ≈400 chips, each patterned with three Si_3_N_4_ membranes (3 mm × 0.7 mm). Hole arrays with gradually changing dimensions are lithographically fabricated on each membrane. Photographs of individual chips before (b) and after Au deposition (c). d) Scanning electron microscopy (SEM) image of an Au‐coated hexagonal lattice MHA with geometrical parameters P_x_ = 5.94 µm, P_y_ = 10.70 µm, and r = 1.57 µm. e) Photograph of a gradient plasmonic MHA metasurface chip, which includes 30 different metasurfaces (MS_1_…, MS_30_) with varying MHA geometric parameters. f) Cross‐sectional view of a single hole showing ≈396 nm Si_3_N_4_ and ≈118 nm Au layer thicknesses, as well as isolated Au nanostructures that are formed due to glancing angle deposition effect on the sidewalls of the holes. g) Au‐coated, gradient MHA patterned, three parallel Si_3_N_4_ membranes on each sensor chip support the excitation of a comb of plasmonic extraordinary transmission resonances. Each metasurface exhibits a unique plasmonic resonance peak in transmission, uniformly covering a spectral range of 1200 to 2000 cm^−1^ in the mid‐IR fingerprint spectrum.

Here, we present a transformative SEIRAS platform that circumvents the fundamental limitations through key innovations in a single device. First, we demonstrate wafer‐scale, high‐throughput fabrication of plasmonic micro‐hole‐array (MHA) metasurfaces on free‐standing silicon nitride (Si_3_N_4_) membranes, yielding ≈400 sensor chips per 6‐inch wafer (**Figure** **1**a). Second, our SEIRAS platform enables transmission mode optical readout because the MHA resonance mechanism is driven by extraordinary optical transmission (EOT), where the interaction between dark surface plasmon polaritons and bright localized surface plasmon modes results in a spectral peak in transmission.^[^ ^37^ ^]^ Third, our gradient MHA design generates a dense spectrum of resonance modes (>400 distinct modes), enabling on‐resonance fingerprint retrieval across the 1200–2000 cm^−1^ fingerprint region. We validated our platform using a model polymer system (poly(methyl methacrylate) (PMMA)), highlighting the critical impact of resonance detuning on SEIRAS‐retrieved absorbance band profiles. Moreover, we analyzed human peritoneal fluid samples from an ovarian cancer patient and an individual with no cancer diagnosis, demonstrating the real‐world utility of our SEIRAS approach in resolving compositional variations by specifically capturing the complex molecular signatures in biological specimens. Overall, this work introduces a practical solution for implementing SEIRAS in real‐world applications, offering a scalable platform for detailed molecular analysis of biological samples. The combination of high‐throughput fabrication, comprehensive spectral coverage, and a simple transmission‐mode optical readout represents a significant advance toward making SEIRAS a viable tool for medical diagnostics and biomarker discovery.

## 2 Results

### 2.1 High‐Throughput Fabrication of Gradient Plasmonic Micro‐Hole‐Array (MHA) Metasurface Chips

The high‐throughput wafer‐scale fabrication of plasmonic MHA gradient metasurfaces begins on a 6‐inch (150 mm) silicon (Si) wafer. First, a ≈400 nm Si_3_N_4_ layer is deposited using low‐pressure chemical vapor deposition (LPCVD), followed by the application of an antireflective coating (ARC) and a UV‐sensitive positive photoresist. Next, the gradient MHA pattern is generated by standard photolithography (using a 365 nm wavelength), and the holes were etched into the Si_3_N_4_ layer using reactive ion etching (RIE) (Figure 1a). To release the patterned Si_3_N_4_ membranes, backside of the wafer was lithographically patterned, and the Si was etched away from the membrane areas. Each MHA chip features three suspended Si_3_N_4_ windows, each measuring a 3.0 mm × 0.7 mm area (Figure 1b). Approximately 400 sensor chips are retrieved from each fabricated wafer. Further fabrication details are included in the methods section and in.^[^ ^38^ ^]^ Once the fabrication of the wafer is complete, individual chips measuring 5.4 mm × 5.4 mm are separated and coated with a ≈100 nm‐thin gold (Au) layer to induce the plasmonic effect (Figure 1c). A scanning electron microscope (SEM) image of the fabricated MHA with a hexagonal lattice is shown in Figure 1d. Figure 1e presents a photograph of the gradient MHA metasurface, which consists of 30 hexagonal hole‐array patterns with varying geometric parameters (P_x_, P_y_, r), labeled MS_1_ through MS_30_ on a single chip. The side view of the plasmonic MHA gradient metasurface (Figure 1f; Figure S1, Supporting Information) shows the cross‐sectional view of a single hole, revealing *a* ≈396 nm Si_3_N_4_ membrane that provides robust mechanical support to a ≈118 nm Au layer, and isolated Au nanostructures formed on the hole sidewalls due to the glancing angle deposition effect. The patterned free‐standing membrane is supported by a 310 µm‐thick Si frame, which is not visible in the SEM image. Upon illumination with a broadband mid‐IR light source, each of the 30 metasurfaces exhibits a resonance peak in transmission, covering the range of 1200 cm^−1^ to 2000 cm^−1^, as shown in Figure 1g.

### 2.2 Plasmonic Resonance Properties of MHA Metasurfaces

**Figure 2:**
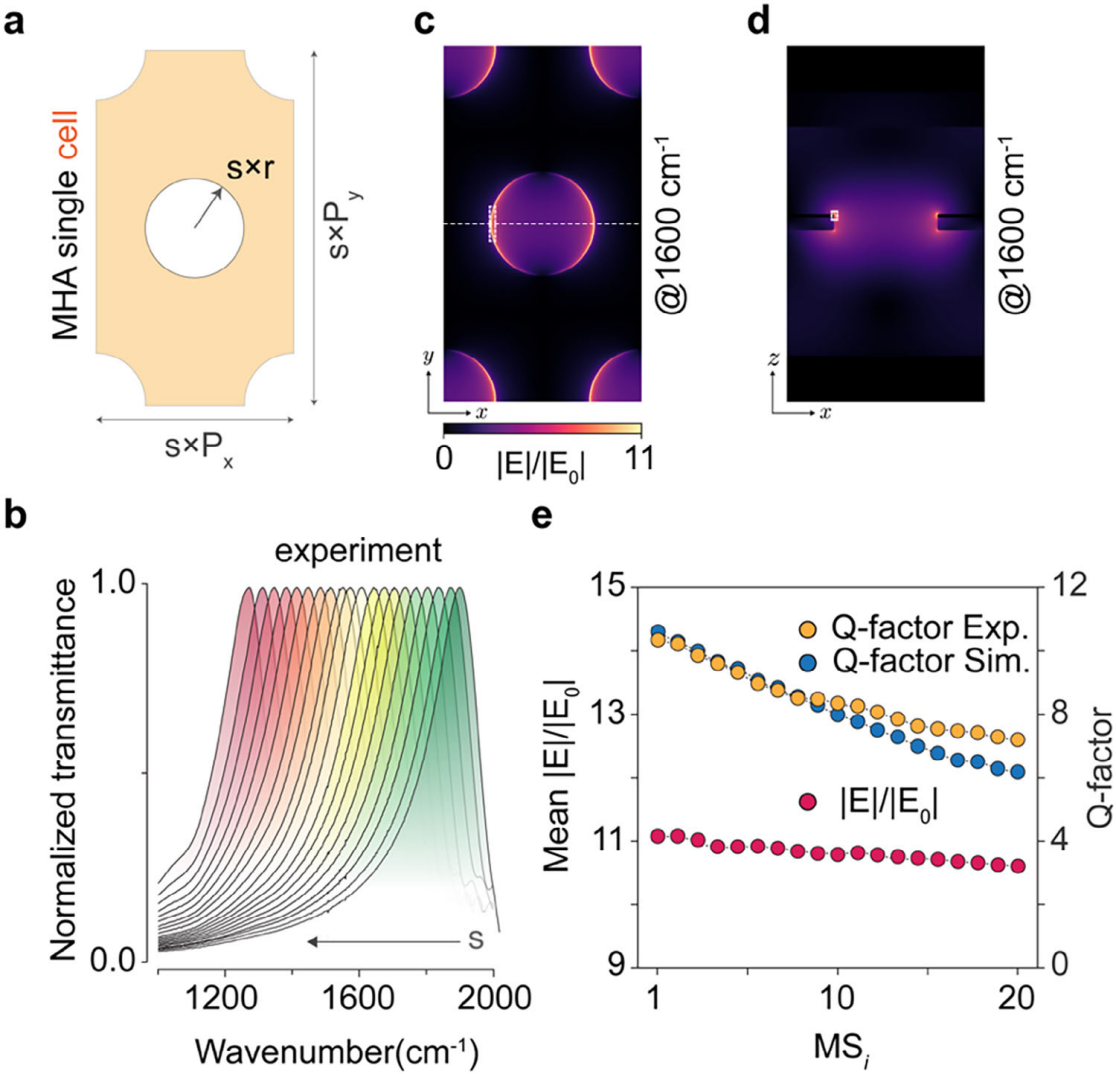
Photonic resonance characteristics of the gradient MHA metasurface. a) Schematic of an MHA unit cell used in simulation to generate a hexagonal lattice with base periods P_x_ = 5.94 µm and P_y_ = 10.70 µm, and a hole radius r = 1.57 µm. These parameters are scaled to sweep the resonance across the desired spectral range. b) Measured transmission spectra of a gradient MHA metasurface. By adjusting the scaling factor S in (a), we tune the wavenumber of the resonance peak from 1200 cm^−1^ (S = 1.38) to 2000 cm^−1^ (S = 0.8). Electric field enhancement, |E|/|E_0_|, maps of the top (c) and side (d) views of a unit cell at the resonance wavenumber (λ_*res*_ =  1600 cm^−1^) showing strong field localization around the rims of the holes. The calculated effective mode volume at the resonance peak is *V_eff_* = 0.0022(λ/*n_eff_*)^3^ with *n_eff_* = 3.033. e) Metasurface resonance frequency dependent Q‐factor variation calculated from experimentally measured (yellow) and simulation‐derived (blue) resonance spectra. The metasurface‐dependent E‐field enhancement is determined by averaging |E|/|E_0_| over the volume indicated by the dashed rectangular regions shown in (c) and (d).

The plasmonic MHA metasurfaces exhibit the distinctive extraordinary optical transmission (EOT) phenomenon^[^ ^37^ ^]^ driven by the interplay of two resonance mechanisms that produce an asymmetric Fano‐type spectral line profile in transmission. When the MHAs are illuminated normally, in‐plane surface plasmon polariton (SPP) modes are excited, satisfying the Bragg's coupling condition through wavevector matching with the grating's momentum. This SPP dark mode interacts strongly with the subwavelength holes in the Au film, generating localized surface plasmon (LSP) modes that scatter light into free space. The near‐field interactions between the dark SPP and bright LSP modes are highly dispersive, giving rise to the Fano‐type transmission peak in the far‐field intensity spectrum (**Figure** **2**; Figures S2 and S3, Supporting Information).

The EOT resonances can be spectrally tuned by modulating the unit cell geometrical parameters, i.e., period along the x‐axis (P_x_ = 5.94 µm), period along the y‐axis (P_y_ = 10.70 µm), as well as the hole radius (r = 1.57 µm), using a scaling factor (S) (Figure 2a). In our gradient MHA metasurface, these EOT resonances sweep the mid‐IR fingerprint region with a dense comb of transmission peaks (Figure 2b). This is achieved by varying the S‐factor from 1.38 to 0.8 over a length of 9 mm to cover a spectral range of 1200–2000 cm^−1^. Overall, the measured transmittance response is in excellent agreement with the simulation (see Figure S4, Supporting Information); the raw spectral data from all metasurfaces exhibit greater than 75% peak transmittance. Moreover, from the electric field maps (Figure 2c top face, and Figure 2d, transversal view), we see that the LSP mode is tightly bound to the rims of the holes, extending into the accessible hole openings (Figure 2d) with a maximum electric field enhancement of |E|/|E_0_|∼11 at 1600 cm^−1^. Figure 2e shows the resonance quality factor (Q‐factor) and the electric field enhancement (mean |E|/|E_0_| from a volume of 16 × 10^−3^ µm^3^) variations in the gradient MHA metasurfaces. When comparing the simulated and experimental responses of the MHA gradient, a decrease in the Q‐factor (≈36% across 20 MS) is observed with increasing resonance wavelength (increasing S‐parameter). Similarly, the E‐field enhancement decreases slightly (≈10% across 20 MS), enabling stable sensing across the fingerprint spectrum.

**Figure 3:**
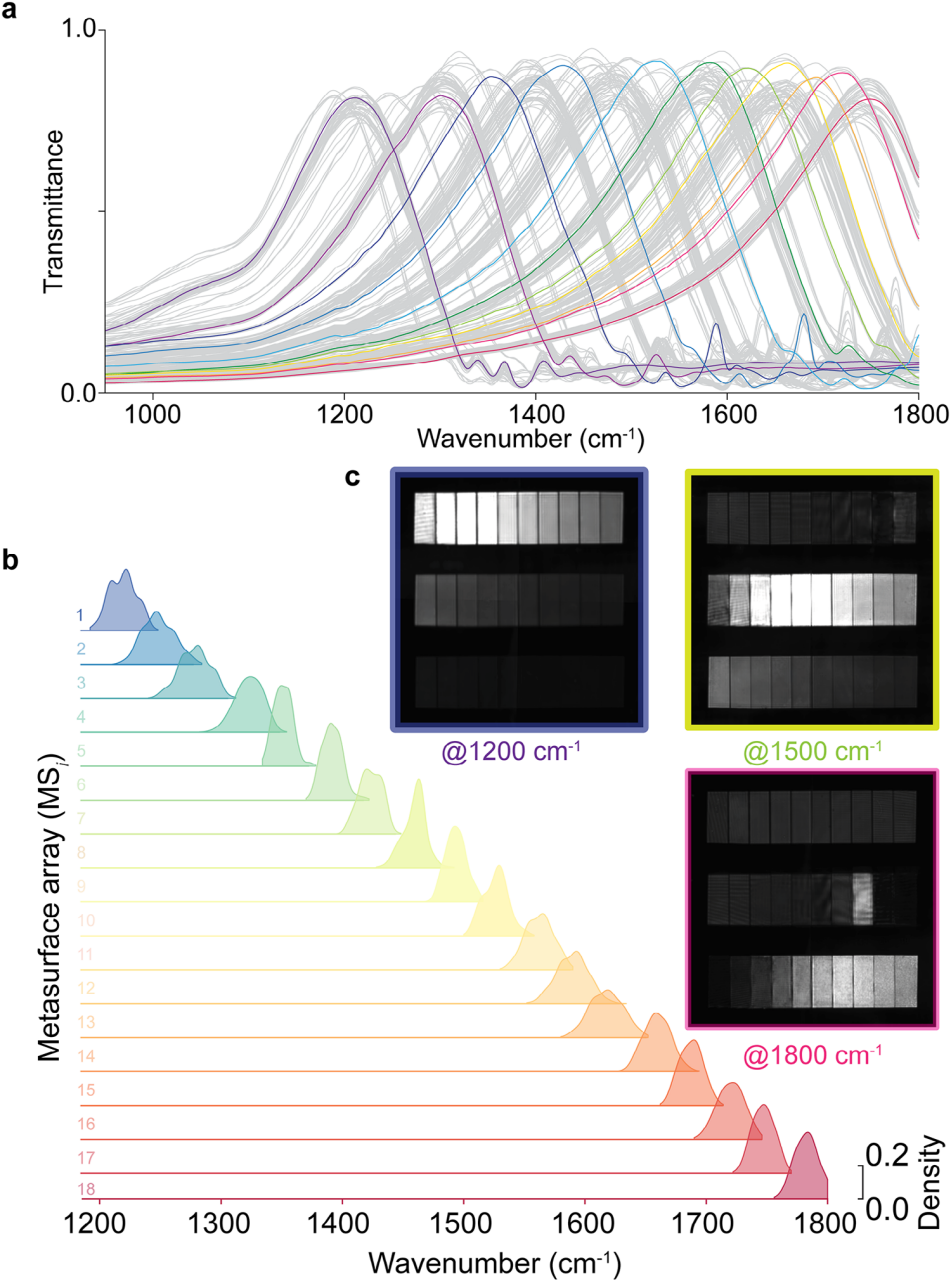
Spatial distribution of resonance peak positions in the gradient MHA metasurface. a) The transmittance plot shows raw pixel spectra from 18 different metasurfaces, exhibiting hundreds of distinct resonances that collectively provide a uniform coverage across the target spectral range. b) Hyperspectral imaging‐based optical interrogation was employed to analyze the pixel‐level density functions of spatial resonance peak distributions across 18 metasurfaces (each sampled by 200 × 200 pixels). c) Mid‐IR transmission microscopy images of the MHA acquired at 1200, 1500, and 1800 cm^−1^. Each rectangular metasurface pattern measures 3.0 mm × 0.7 mm.

We further investigated the spatial variation of the resonance properties in the gradient MHA metasurfaces using a mid‐IR hyperspectral imaging microscope. Coupled with a tunable quantum cascade laser (QCL), we collected hyperspectral image data cubes by sweeping collimated, normally incident, and linearly polarized mid‐IR light between 5.5 and 10.5 µm. From each metasurface area, we considered 200 × 200 pixel‐spectra and plotted their resonance wavelengths on a density plot (**Figure** **3**). We measured minimum and maximum standard deviations of 7 and 13 cm^−1^, respectively, for gradients MS_1‐18_. From the density plot, we observed that our fabricated gradient MHA metasurfaces densely populate the spectral region of interest, with at least 20 wavenumbers of overlap in pixel‐resonance distribution between adjacent metasurfaces. This overlap is especially advantageous for retrieving vibrational bands with minimal distortion, as will be discussed in the next sections. Additionally, Figure 3 shows three mid‐IR transmission microscopy images of the MHA gradient chip, revealing three parallel patterned membrane regions. These distinct regions light up sequentially from top to bottom, in accordance with their resonance position, as the illumination wavenumber changes (1200, 1500, and 1800 cm^−1^).

### 2.3 On‐Resonance SEIRAS Using Plasmonic Gradient MHA Metasurfaces

**Figure 4:**
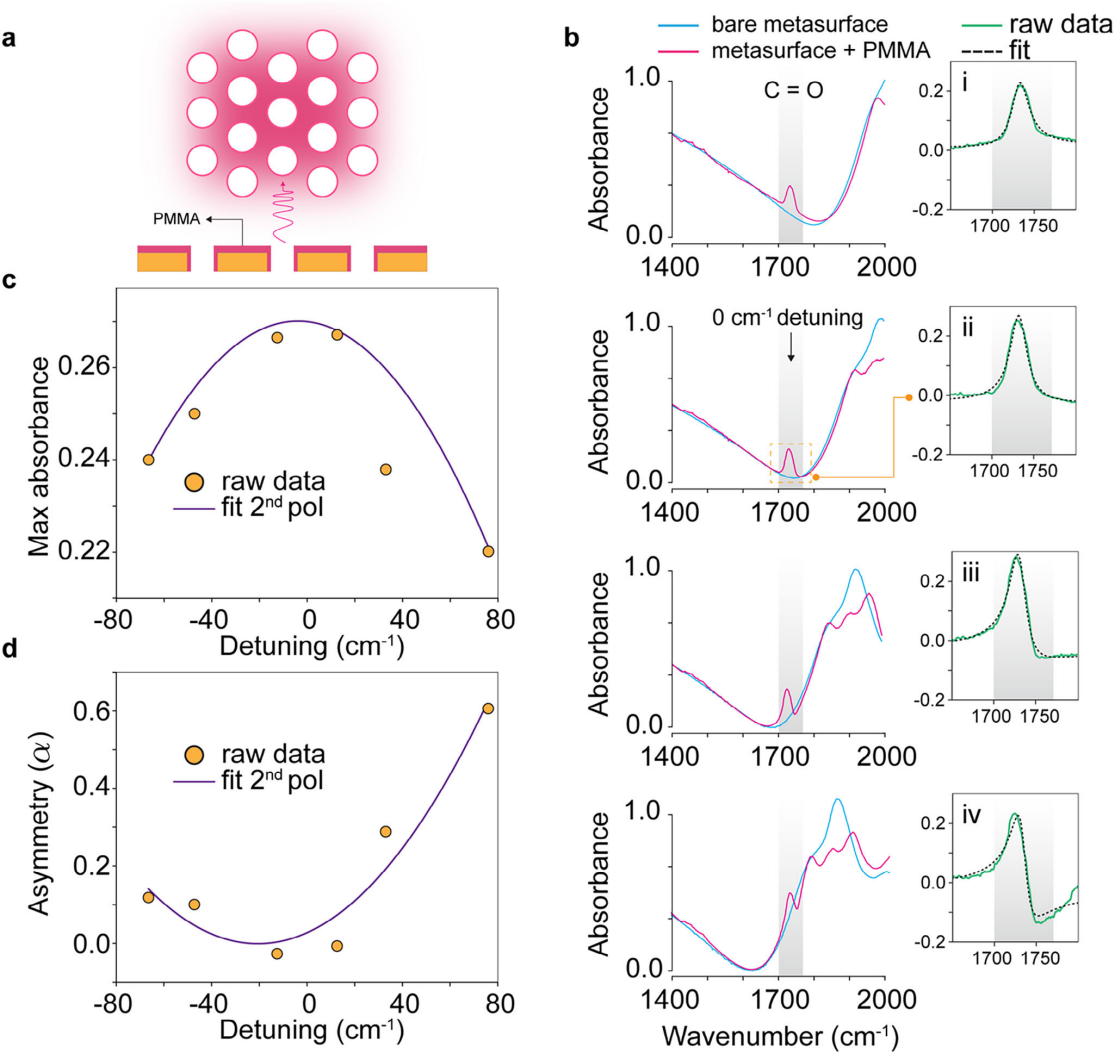
On‐resonance SEIRAS with gradient plasmonic MHA metasurfaces. a) A 70 nm‐thick PMMA layer was spin‐coated on the top face of the gradient MHA metasurface. b), Measured absorbance spectra of metasurfaces with varying resonances before (blue) and after (pink) PMMA coating. The insets show the retrieved PMMA absorbance band obtained by subtracting the bare metasurface spectra from those of the PMMA‐coated metasurface. As the bare resonance of the metasurface detunes from the 1730 cm^−1^ C═O vibrational band of PMMA molecules, the absorbance profile deviates from Gaussian to Fano‐like shape. c,d) Calculated maximum absorbance (c) and Fano factors (d) from the SEIRAS retrieved C═O absorbance bands in (b) indicate that, at zero detuning (b‐ii), the C═O absorbance band is highly symmetric, highlighting the importance of gradient metasurfaces in SEIRAS for accurately detecting realistic absorption band profiles.

To demonstrate the critical role of on‐resonance SEIRAS for chemical characterization of complex compounds, we investigated the effects of spectral proximity between the plasmonic resonance and the carbonyl vibrational band (C═O at 1730 cm^−1^) of PMMA molecules. We began by measuring the spectral response of a bare MHA gradient metasurface to establish a baseline. Subsequently, a 70 nm‐thick PMMA layer was spin‐coated onto the top face of the same gradient MHA chip, as artistically rendered in **Figure** **4**a. Figure 4b shows the measured absorbance spectra of the coupled system, comprising the EOT plasmonic resonance and the carbonyl band of PMMA, from multiple MHA metasurfaces. The response of this coupled system matches with our simulation results (Figure S5, Supporting Information), where we report on‐resonance SEIRAS signals of the carbonyl band with increasing PMMA layer thickness, showing quantitative sensing capabilities of our method. To extract the metasurface‐enhanced absorbance spectra of the carbonyl band, we subtracted the transmittance baseline of the bare MHA from that of the MHA‐PMMA coupled system (Figure 4b insets). When the MHA resonance peak is finely aligned with the carbonyl vibrational mode (Figure 4b–ii), the retrieved absorption band displays an almost symmetrical shape and fits well to a Gaussian curve. In contrast, when the resonance peak is detuned either to the left or right of 1730 cm^−1^, the retrieved carbonyl band becomes asymmetric, exhibiting a Fano‐like profile (Figure 4b–i, iii, iv). To quantify detuning effects, we calculated the degree of asymmetry using a Fano fit (details in Experimental Section) and plotted the asymmetry factor (α) as a function of detuning (Figure 4d). We also calculated the maximum absorbance as a function of spectral detuning (Figure 4c), and, as expected, found that the maximum enhancement occurs at zero detuning point, where the coupling is highest. This experiment demonstrates not only the impact of the resonance peak alignment on accurately and sensitively retrieval of the vibrational bands but also highlights the importance of our SEIRAS approach using gradient metasurfaces for precise chemical analysis of complex samples.

**Figure 5:**
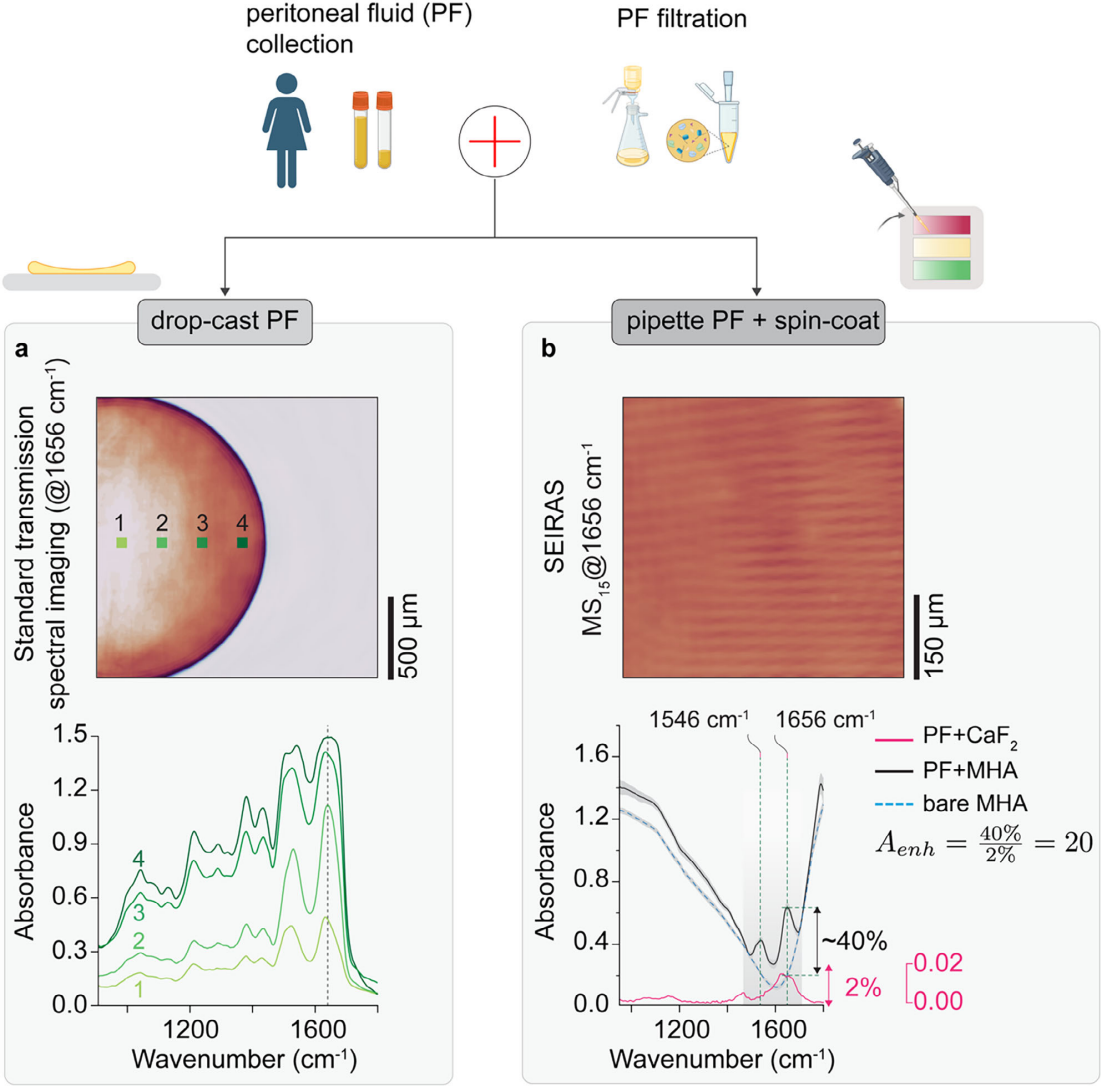
Mid‐IR spectral analysis of human bodily fluids. a) Mid‐IR transmission microscopy image of drop‐casted, dried peritoneal fluid on a standard CaF_2_ substrate, captured at the Amide I protein band (1656 cm^−1^) using a hyperspectral imaging instrument. The absorption image reveals uneven PF sample thickness due to the coffee‐ring effect. Spectra from four different spots along the radial axis of the droplet (1,2,3, and 4) exhibit drastic quantitative and qualitative variations in the absorbance fingerprints, despite originating from the same sample. b) Mid‐IR transmission microscopy image of the spin‐coated PF sample on a metasurface, which exhibits uniform absorbance unlike the drop‐casted PF. In the bottom absorbance spectra, the black line represents the mean value of the SEIRAS measurements collected from a metasurface area of 90 × 10^3^ µm^2^, while the shaded gray region corresponds to the standard deviation. The green dashed line shows the bare metasurface spectrum from the identical area, which is used as a reference to extract total SEIRAS absorbance (40% for the Amide band at 1656 cm^−1^). The pink spectrum is collected from spin coated PF on standard CaF_2_ substrate, showing 2% absorbance at the 1656 cm^−1^ protein band. The plasmonic metasurface enhances the absorbance signals ≈20‐times, enabling the access to consistent fingerprint spectra of complex PF samples.

An important application field for vibrational spectroscopy is biomedicine, where non‐targeted, label‐free chemical analysis of complex biological samples is required. However, in real‐world samples, accurate identification of spectral fingerprints in the mid‐IR region suffers from spectral congestion and non‐specific variations due to measured samples’ physical features.^[^ ^9^ ^]^ As an example, we analyzed a human peritoneal fluid (PF) sample from an ovarian cancer patient (see Experimental Section) by drop casting a 5 µL of PF on a standard CaF_2_ substrate. **Figure** **5**a shows a mid‐IR absorption image captured at the Amide I band (1656 cm^−1^) using a standard transmission‐mode measurement. Herein, we demonstrate the nonuniform PF sample distribution forming a coffee‐ring stain as it dries on the CaF_2_ surface. In Figure 5a, we further plot the absorbance spectra from four different spatial positions (1,2,3, and 4) of the same sample. Spectra from positions 1 and 2 do not saturate because these regions are thinner, yet the weak bands are not accurately captured. By contrast, the spectra from thicker regions saturate the strong amide bands, and the Mie scattering generates a large baseline.^[^ ^10^ ^]^ Overall, this experiment demonstrates that the spectral fingerprints exhibit distinct quantitative and qualitative differences, despite being measured from the same PF sample, highlighting the challenges of accurate spectral measurements of real‐world samples.

For accurate chemical analysis of complex biological samples, we developed gradient metasurfaces whose plasmonic resonances continuously sweep the mid‐IR spectral range, enabling on‐resonance SEIRAS across the fingerprint region. Our method begins with spin‐coating a 50 µL of PF at 4000 rpm onto the top surface of the MHA gradient metasurface (see Experimental Section). Figure 5b presents a mid‐IR absorption image of thinly coated PF on the MHA metasurface, recorded in transmission mode at the Amide I band (1656 cm^−1^). We further processed the spectra from 200 × 200 number of pixels in this region, plotting their absorbance mean and standard deviation across the fingerprint region (Figure 5b). The absorption bands captured across the metasurface remain quantitatively and qualitatively uniform, highlighting the strength of SEIRAS in characterizing thinly spread complex biological samples.

To quantify the enhancement in absorption signals due to our MHA metasurfaces, we followed the same protocol and spin‐coated PF onto a standard CaF_2_ substrate (pink spectrum in Figure 5b; Figure S6, Supporting Information). While the most prominent protein band at 1656 cm^−1^ shows only ≈2% amplitude on CaF_2_, the same quantity of analyte on the plasmonic metasurface (PF + MHA) produces a ≈40% absorbance for the same band. This translates into an effective absorbance enhancement of 20‐fold. Importantly, this enhancement extends beyond signal amplification to improved spectral quality. The spectrum from spin‐coated PF on bare CaF_2_ exhibits higher noise levels and fails to resolve weaker bands, e.g., ≈1546 cm^−1^. In contrast, the PF fingerprint spectrum retrieved from the SEIRAS is significantly smoother because the enhanced spectral peak values remain well above the noise floor of the IR detectors.

**Figure 6:**
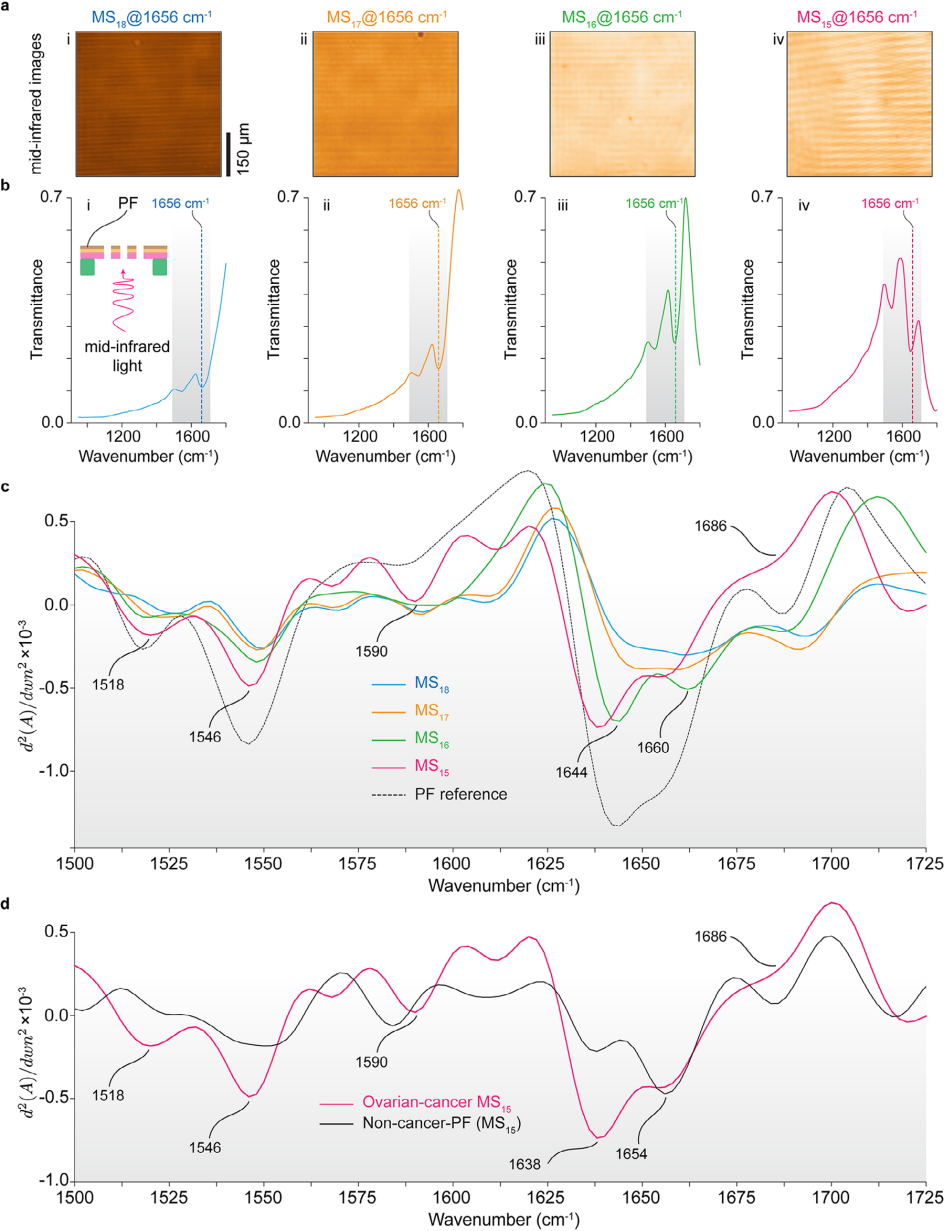
Spectrochemical analysis of complex peritoneal fluids using the plasmonic gradient metasurfaces. a) Mid‐IR transmission microscopy images of the MHA metasurfaces, MS_18_, MS_17_, MS_16_, and MS_15_, spin‐coated with a thin layer of PF on the top face. Changes in the transmittance intensity at 1656 cm^−1^ are due to differences in the metasurface resonance positions. b) Measured transmittance spectra of PF‐coated metasurfaces, whose resonances sweep the amide I and II regions (highlighted in gray). The absorbance enhancement becomes more prominent when the protein‐associated amide bands are tuned to the resonance peaks. c) Second derivative analysis shows that sub‐vibrational bands are better resolved when they are aligned with the metasurface resonance, exhibiting minimal distortion in their band profiles compared to the PF reference signal. d) Comparison of measured second‐derivative spectra of PF samples from an individual with no cancer diagnosis (“Non‐cancer‐PF (MS_15_)”) and an ovarian‐cancer patient (Ovarian‐cancer MS_15_), both measured from MS_15_.

Using the same rationale as in our coupled MHA‐PMMA detuning study, we investigated how the detuning affects the SEIRAS response of a complex human PF sample. Specifically, we compared spectra from four adjacent metasurfaces (MS_18_, MS_17_, MS_16_, and MS_15_) whose resonances sweep through the protein‐associated amide I and II spectral regions (gray shaded areas in **Figure** **6**). Figure 6a shows mid‐IR transmittance images of the four metasurfaces spin‐coated with PF, captured at the amide I band (1656 cm^−1^). We also plotted the average transmittance spectra from these images in Figure 6b. Vibrational bands associated with functional groups in biomolecules distort the plasmonic EOT resonances, suppressing transmittance peak in the far‐field. We observed that these vibrational bands appear asymmetric when they are not spectrally aligned with the resonance peak of the plasmonic MHA metasurfaces, similar to the behavior seen in the MHA‐PMMA coupled system. Moreover, because the PF exhibit more complex bands than those of the PMMA molecules, we analyzed the PF fingerprint spectra by taking the second derivative of its absorbance signals from the coupled MHA‐PF system. Figure 6c compares these second‐derivative spectra from four adjacent MHA metasurfaces with the reference spectrum of the same PF sample collected using a standard transmission‐mode measurement.

Our second‐derivative analysis revealed that when molecular absorption bands are probed with on‐resonance SEIRAS, their amplitudes and spectral positions are better resolved than in the detuned cases. For example, vibrational bands ≈1518, 1546, and 1590 cm^−1^ are accurately retrieved by the MS_15_ (Figure 6c, magenta) and their spectral positions show good agreement with the reference signal collected from bulk PF sample on CaF_2_ substrate (Figure 6c, gray). In contrast, these bands appear weak in the spectra from the adjacent metasurfaces (MS_16,_ MS_17,_ MS_18_) and their spectral positions vary due to the asymmetry introduced by resonance detuning. Likewise, the bands of 1644, 1660, and 1686 cm^−1^ (Figure 6c, green) are better captured by MS_16_. Moreover, our measurements unveil how SEIRAS addresses the issue of spectral congestion in standard mid‐IR spectroscopy. For instance, the two closely spaced bands (1644 and 1656 cm^−1^) are barely distinguishable in the gray reference spectra in Figure 6c, while they are well separated in measurements with MS_15_ and MS_16_. This occurs because, in bulk sample measurements, the strong absorption from abundant molecules overshadows weaker bands. Notably, SEIRAS minimizes spectral congestion because the measurement volume is confined to plasmonic hotspots, where a small quantity of molecules can generate a high signal‐to‐noise ratio absorption signal due to enhanced light‐matter interactions. Thus, our MHA metasurface enables superior qualitative analysis of fingerprint spectra by providing enhanced spectral discrimination of neighboring bands in congested spectral regions (e.g., protein bands). Additionally, quantitative analysis of analytes using the MHA metasurface can be achieved by measuring the amplitude of the dips in resonance transmittance peak of the metasurface (see Figure S7, Supporting Information).

Lastly, we compared the PF fingerprint spectra from an individual with no cancer diagnosis (“Non‐cancer‐PF”) and from an ovarian‐cancer patient in Figure 6d. Significant deviations in the second derivative spectra underscores the diversity of compositional variations in each sample. While preliminary spectral differences are evident, it is beyond the scope of this paper to comment on the spectral signatures that can potentially be associated with cancer patients. Our results set a solid ground for future biomarker investigations through clinical studies including a large cohort of samples combined with ML/AI‐based classifiers.

## 3 Discussion

In this work, we present several key advances in SEIRAS technology for analyzing complex biological samples, establishing a solid workflow ready for future clinical studies. By fabricating plasmonic gradient metasurfaces on free‐standing Si_3_N_4_ membranes using wafer‐scale processes, we achieved an unprecedented manufacturing throughput (≈400 chips per 6″ wafer). The scalable fabrication of plasmonic metasurfaces was possible because our approach does not require IR‐transparent substrates (e.g., CaF_2_ and MgF_2_), which are not compatible with wafer‐scale processes. Instead, we leveraged free‐standing Si_3_N_4_ membranes, which are mechanically robust, compatible with standard semiconductor processing, and spectrally transparent in the molecular fingerprint region. This advancement addresses the critical challenge of low throughput metasurface fabrication and makes SEIRAS technology practically viable for widespread adoption in real‐world sensing applications.

Second, our transmission‐mode operation simplifies the optical readout setup, enabled by the high transmission efficiency (T > 75%) EOT modes of the plasmonic MHA metasurfaces. Our sensor chips allow optical interrogation using robust, collinear imaging platforms consisting of a broadband IR light source, a microbolometer IR camera, and simple lenses, as previously demonstrated by Meng et al.^[^ ^40^ ^]^ Such straightforward and compact analytical platforms can further broaden the impact of SEIRAS by facilitating its implementations as on‐site sensors for real‐time monitoring, and quantitative molecular sensing.

Third, our method addresses a well‐known challenge in SEIRAS – capturing broadband fingerprint spectra of complex samples containing unknown bands. To overcome this limitation, we implemented a rainbow metasurface design featuring spatially encoded resonances that systematically sweep across the target spectral range. This approach ensures that any unknown absorption band will be well‐aligned with resonances from a specific region of the metasurface. Consequently, all vibrational bands within the range of 1200 to 2000 cm^−1^ are continuously probed by multiple overlapping resonances, leading to accurate and sensitive retrieval of fingerprint spectra from complex biological samples.

Additionally, to further optimize the interaction between plasmonic and vibrational bands of unknown samples, the measured SEIRAS signals can be fitted to a Fano line‐shape. By assessing the asymmetry of the SEIRAS signals across different metasurface pixels, regions generating the most symmetric bands can be identified, ensuring optimal on‐resonance conditions. We demonstrated this approach to identify the most accurate line‐shape of the on‐resonance PMMA band in Figure 3. Thus, our gradient metasurface design ensures on‐resonance SEIRAS across the fingerprint region, which is particularly important when analyzing complex biological samples containing multiple unknown bands.

To benchmark our SEIRAS platform against a typical SEIRAS setup, we fabricated new metasurfaces consisting of hexagonal Au‐pillar arrays on a CaF_2_ substrate. For an unbiased comparison, we designed the inverse of the MHAs by creating Au pillars instead of holes, while keeping the geometrical parameters identical. These comparative measurements demonstrate that our MHA metasurface approach outperforms conventional plasmonic metasurfaces in capturing SEIRAS signals in transmission mode (see Figure S8, Supporting Information). The enhanced performance of the MHA is primarily due to their high transmittance at resonance, resulting in many photons reaching the detector at the peak maximum (T > 75%), which is way above the detector's noise floor.

Finally, we demonstrated our technology's real‐world utility by successfully analyzing peritoneal fluid samples from ovarian cancer patients and individuals with no cancer diagnosis. Conventional IR spectroscopy face inherent trade‐offs when analyzing complex biological samples, compromising spectral quality. For example, when bulk sample quantities are deposited on standard CaF_2_ substrates and dried, variations in sample thickness and composition, combined with optical scattering effects, distort and congest the resulting spectra. While spin‐coating can produce uniform thin sample layers, it generates absorption signals too weak for accurate spectral fingerprint retrieval. Our SEIRAS method overcomes these issues by delivering accurate and robust fingerprint spectra from compositionally complex biological samples, offering unprecedented opportunities in medical diagnostics and biological research.

In sum, we addressed the key limitations of traditional SEIRAS methods through innovative design and fabrication approaches, representing a significant step toward practical, high‐throughput, and compact biochemical sensors. The combination of scalable fabrication of plasmonic chips, simplified imaging‐based optical readout in transmission mode, and the capability to characterize complex biological samples establishes a foundation for translating SEIRAS technology into routine medical diagnostic and biomedical research applications.

## 4 Experimental Section

### Numerical Simulations

The finite‐element frequency‐domain solver (CST Microwave Studio 2023, Dassault Systèmes, France) was used to calculate the transmittance spectra in Figures 1g, and 2e. The electric field enhancement, depicted in Figure 2c,d, was calculated using Tidy3D FDTD (Flexcompute, California, USA). In all simulations, periodic boundary conditions were applied along the x‐ and y‐axes, while perfectly matched layers (PML) were used along the z‐direction. Plane wave illumination was assumed with a Gaussian temporal profile, incident normally from the top of the structure. The optical constants for Au and Si_3_N_4_ were taken from references,[^41^, ^42^] respectively. The cavity's effective mode volume was calculated as described in reference.^[^ ^43^ ^]^

(1) $$V_{eff}=\frac{\int \varepsilon \left(r\right){\left|E\left(r\right)\right|}^{2}d^{3}r}{\max\left(\varepsilon \left(r\right){\left|E\left(r\right)\right|}^{2}\right)}$$

### Device Fabrication

The gradient MHA metasurfaces were fabricated on a 150 mm Si wafer, 310 µm thick 〈100〉 p‐type, doubled‐side‐polished Si wafers. A 400 nm Si_3_N_4_ layer was deposited using LPCVD onto both the top and bottom faces of the wafer. This was followed by the application of an ARC and a UV‐positive photoresist onto the wafer's front side. After conventional photolithography (365 nm wavelength) patterning, the MHA gradient geometry was transferred into the ARC via Ar/O_2_ plasma etching and subsequently into the Si_3_N_4_ layer using reactive ion etching with SF_6_ and He. To serve as a protective layer, a 200 nm SiO_2_ coating was deposited over the MHA‐patterned Si_3_N_4_.

The backside of the wafer was then patterned with a mask to define the chip's outer frame dimensions (5.4 mm × 5.4 mm) and three free‐standing suspended membrane windows (3.0 mm × 0.7 mm). Finally, the bulk Si was removed using wet etching. Further details of the fabrication process can be found in reference.^[^ ^38^ ^]^

### PMMA Spin Coating and Thickness Characterization

A 70 nm‐thick PMMA layer (PMMA 950 A2, Kayaku Advanced Material, Massachusetts, USA) was deposited using spin coating at 4000 rpm. The metasurface chip was then baked at 180 °C for 5 min. To measure the PMMA thickness, each metasurface coated with PMMA was accompanied by a bare Si chip of the same size, which was also coated with PMMA under identical conditions. The thickness of the PMMA layer on the bare Si substrates was measured using an ellipsometer (J.A. Woollam, Nebraska, USA) and a reflectometer (Filmetrics, California, USA).

### Peritoneal Fluid Coating

Human PF was collected from patients with high‐grade serous ovarian cancer as part of their standard‐of‐care treatment. These studies were approved by the Institutional Review Board (IRB), and all patients provided informed consent prior to PF collection. The samples were stored at –80 °C within 2 h of collection. To minimize aggregates in suspension—which could rupture the membrane due to mechanical stress during drying—the freshly collected PF was filtered using a Corning 50 mL vacuum filter with a 0.22 µm pore size. The absorbance spectrum was measured both before and after filtration. No significant change was observed in the bulk absorbance spectrum profile between the filtered and non‐filtered PF samples. Next, 50 µL of PF was pipetted onto the top surface of the MHA gradient metasurface and incubated for 5 min at room temperature before being spin‐coated at 4000 rpm. The chip was then left to dry for 5 h at room temperature before being placed in the nitrogen‐purged chamber of the mid‐IR microscope for spectral measurements. For the drop‐casted PF, 5 µL of filtered PF was deposited onto a standard mid‐IR substrate. It was allowed to dry for 5 h at room temperature before mid‐IR measurements were performed.

### Optical Setup and Measurements

Mid‐IR spectral measurements were performed using a tunable QCL integrated into a mid‐IR microscope (Spero‐QT, Daylight Solutions, California, USA), unless otherwise noted. The microscope, equipped with four QCL modules, can collect spectra covering the fingerprint region from 950 to 1800 cm^−1^ with a spectral resolution of 2 cm^−1^. During acquisition, the sample chamber was continuously purged with dry nitrogen to remove water vapor. The free‐standing metasurface was illuminated with collimated, linearly polarized light at normal incidence. Spectral data were acquired in transmission mode using a 12.5× IR collection objective (0.7 NA) and detected using an uncooled microbolometer focal plane array with 480 × 480 pixels, providing a field of view of 650 µm × 650 µm. In transmission mode, the chemical microscope captures the sample's decadic absorbance as:

(2) $$A=-\log_{10}\left(T\right)=-\log_{10}\left(I/I_{\circ }\right)$$

Where *T* = *I*/*I*_○_ represents the transmission through a standard CaF_2_ substrate. From the definition of decadic absorbance, the transmittance of the sample is calculated as:

(3) $$T=10^{-A}\;$$

Figure 2b (experiment) and Figure 4b were composed using data collected with a Fourier‐transform infrared (FTIR) spectrometer coupled to an infrared microscope (Bruker Vertex 70 FTIR and Hyperion 2000). Transmittance spectra were obtained using linearly polarized light and a low‐NA refractive objective (5×, 0.17 NA, Pike Technology, Wisconsin, USA). The setup employed collimated light, achieved by removing the bottom condenser, and a liquid‐nitrogen‐cooled mercury‐cadmium‐telluride (MCT) detector for spectral measurements. All acquired transmittance spectra of the MHA gradient metasurfaces were normalized to the transmittance of standard CaF_2_ windows. While the theoretical limit for the transmittance signal is 1, that is not the case for the case for the decadic absorbance. For example, in Figures 4b and 5, the absorbance signals are greater than 1. In Figure 4b, this occurs because of the resonance spectral characteristics of the MHA, the transmittance spectrum degrades to very low values at the wavenumbers away from the resonance (see raw transmittance spectra in Figure 3). At these off‐resonance wavenumbers, *I*_○_ ≫ *I* in Equation (2). When $\frac{I}{I_{0}}<10^{-1}$, which is feasible for highly absorptive materials, then *A* > 1. Similarly, in Figure 5a, at the amide protein bands (≈1650 cm^−1^), the sample is very thick at the edges of the droplet, resulting in a significant reduction in transmission and thus and absorbance *A* > 1.

### Data processing

The measured transmittance spectrum in Figure 2b (experiment) was normalized to its maximum for visualization purposes. All cavity losses and Q‐factors were determined by fitting the raw transmittance data shown in Figure 2b to the following Fano expression:^[^ ^44^ ^]^

(4) $$T={\left|ie^{i\phi }t_{0}+\frac{\Gamma_{R}}{\Gamma_{R}+\Gamma_{NR}+i\left(\lambda -\lambda_{res}\right)}\right|}^{2}$$

Where *ie* ^*i*ϕ^ *t*_0_ describes the background and the shape of the resonance, with the asymmetry factor α being defined as *t*_0_. The Q‐factor can then be calculated as:

(5) $$Q=\frac{\lambda_{res}}{2\left(\Gamma_{R}+\Gamma_{NR}\right)}$$

Where λ_*res*_, Γ_*R*_, and Γ_*NR*_ are the resonance wavelength, radiative, and absorptive loss rates, respectively.

## Conflict of Interest

The authors declare no conflict of interest.

## Supporting information



Supporting Information

## Data Availability

The data that support the findings of this study are available from the corresponding author upon reasonable request.
