# Supplementary material for: Mass‐Manufactured Gradient Plasmonic Metasurfaces for Enhanced Mid‐IR Spectrochemical Analysis of Complex Biofluids
Source: Adv Mater. 2025 Aug 29;37(47):e04355. doi: 10.1002/adma.202504355 (PMC12535766; doi:10.1002/adma.202504355)
Supplement: Supplementary file 1 — Supporting Information [file ADMA-37-e04355-s001.pdf]

ADVANCED MATERIALS

Supporting Information

for *Adv. Mater.*, DOI 10.1002/adma.202504355

Mass-Manufactured Gradient Plasmonic Metasurfaces for Enhanced Mid-IR Spectrochemical Analysis of Complex Biofluids

*Samir Rosas, Shovasis Kumar Biswas, Wihan Adi, Furkan Kuruoglu, Aidana Beisenova, Manish S. Patankar and Filiz Yesilkoy**

Mass-manufactured Gradient Plasmonic Metasurfaces for Enhanced Mid-IR Spectrochemical Analysis of Complex Biofluids

Samir Rosas[1,‡], Shovasis Kumar Biswas[2,‡], Wihan Adi[1], Furkan Kuruoglu[1,3], Aidana Beisenova[1], Manish S. Patankar[4], Filiz Yesilkoy[1]*

[1] Department of Biomedical Engineering, University of Wisconsin–Madison, Madison, WI 53706, USA

[2] Department of Electrical and Computer Engineering, University of Wisconsin-Madison Madison, WI 53706, USA

[3] Department of Physics, Faculty of Science, Istanbul University, Vezneciler, 34134, Istanbul, Turkey

[4] Department of Obstetrics and Gynecology, University of Wisconsin–Madison, Madison, WI 53792, USA

*Corresponding author. Email: filiz.yesilkoy@wisc.edu

‡ Authors contributed equally

Supplementary Information

This PDF file includes:

Fig. S1-S8

In Figure S1, we present new SEM images showing the cross-sectional views of our device. These views clearly highlight the critical thickness parameters of the silicon nitride and gold layers in the free-standing membrane.

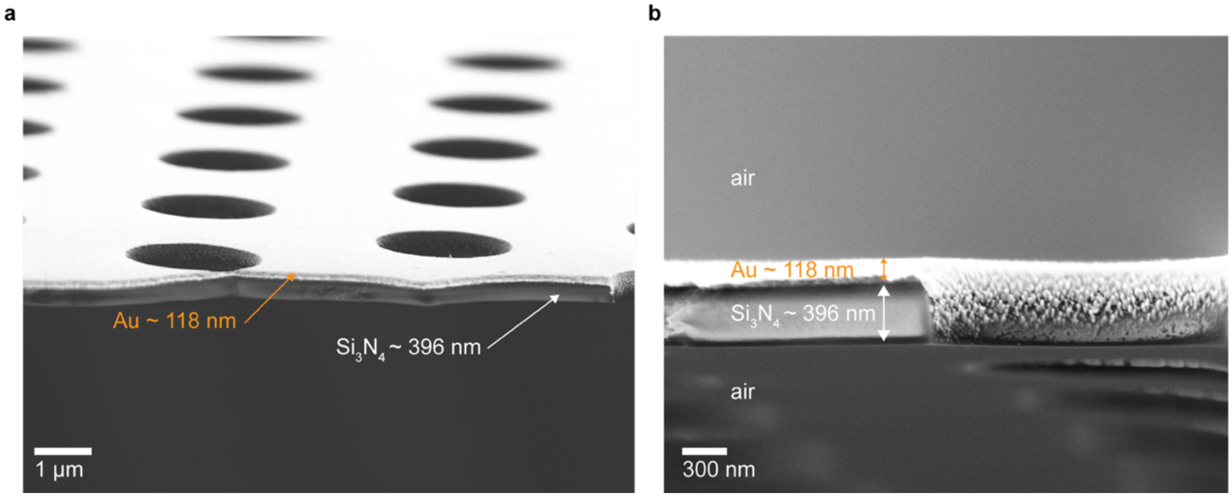

Figure S1. SEM images showing the cross-sectional views of the MHA. a, Cross-sectional SEM views of Au microhole arrays patterned on a free-standing silicon nitride (Si_3N_4) membrane coated with an Au layer. **b**, Cross-sectional view of a single hole showing ~396 nm silicon nitride (Si_3N_4), and ~118 nm Au layer thicknesses, as well as isolated Au nanostructures that are formed due to glancing angle deposition effect on the sidewalls of the holes.

Extraordinary Optical Transmittance (EOT)

Excitation of SPP modes at the interface between an unpatterned Au film and a dielectric medium requires prism based Kretschmann configuration. Yet, in our measurement setup, we use normally incident light using low-NA refractive objectives. Our setup does not satisfy the momentum matching condition to excite SPP modes. Furthermore, we have simulated the transmittance through an unpatterned gold film of ~ 100 nm thickness. This simulation showed negligible transmission in the mid-infrared region (see Fig. S2a, pink line). Second, we reduced the radius of the holes from 1.5 μm to 0.5 μm while keeping all other design parameters constant (see Figure S2a). We present that the amplitude of the transmission signal vanishes with decreasing hole size, confirming that the observed EOT resonances are generated by the MHA structure are a direct result of the triangular hole array.

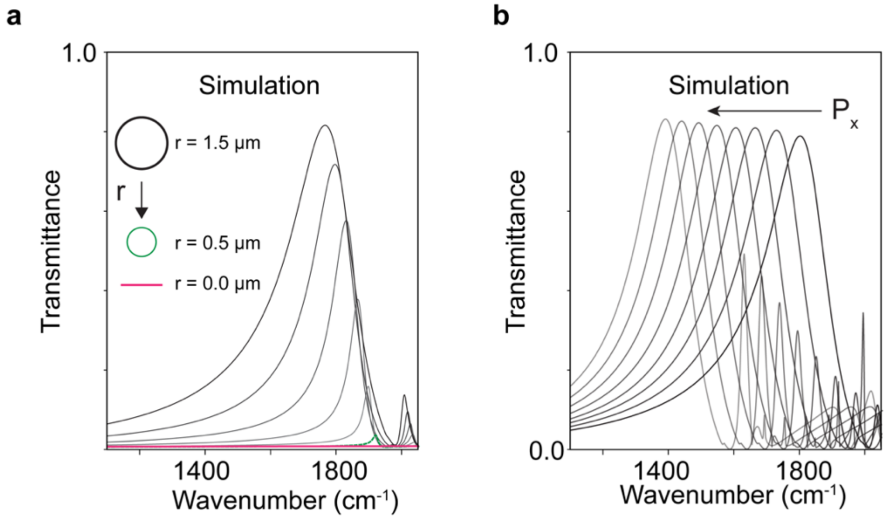

Figure S2. Simulation of the effect of MHA geometrical parameters on the transmittance spectrum. a. As the radius of the holes decreases the amplitude of the mode also decreases. **b.** By modifying the periodicity of the hole array while keeping the radius constant, we can tune the resonance mode positions.

As it is discussed in Genet, C., Ebbesen, T. "Light in tiny holes"[4], periodic triangular hole arrays in a continuous metal film can enable excitation of propagating surface plasmons by compensating the momentum mismatch between the incident photons and SPPs. Thereby, periodic hole arrays we used in our design can give rise to the EOT mode. For example, if we applied the momentum matching condition discussed in Genet, C., Ebbesen, T. Light in tiny holes, to our hole array shown in Figure S3b, we can show that the dark SPP mode position λ_{res} at normal incidence are given by:

$$\lambda_{res} = \frac{P}{\sqrt{\frac{4}{3}(i^2 + ij + j^2)}}\sqrt{\frac{\varepsilon_m \varepsilon_d}{\varepsilon_m + \varepsilon_d}}$$

Where P is the period of the array, ε_m and ε_d are respectively the dielectric constant of the metal and the dielectric material in contact with the metal and i , j are the scattering orders of the array. When we insert our design parameters, $P = 6$ μm, $\varepsilon_m = \text{Real}\,[(4.574 + 42.46i)^2] = -1781.93$ from (Olmon, Robert L., et al. "Optical dielectric function of gold." Physical Review B—Condensed Matter and Materials Physics 86.23 (2012): 235147.[5]) and $\varepsilon_d \approx 1$, we calculate the (1,0) Air-Au mode occurs at $\lambda_{res} = 5.2$ um. This value corresponds to the dark mode we measured in the spectrum of our hole arrays.

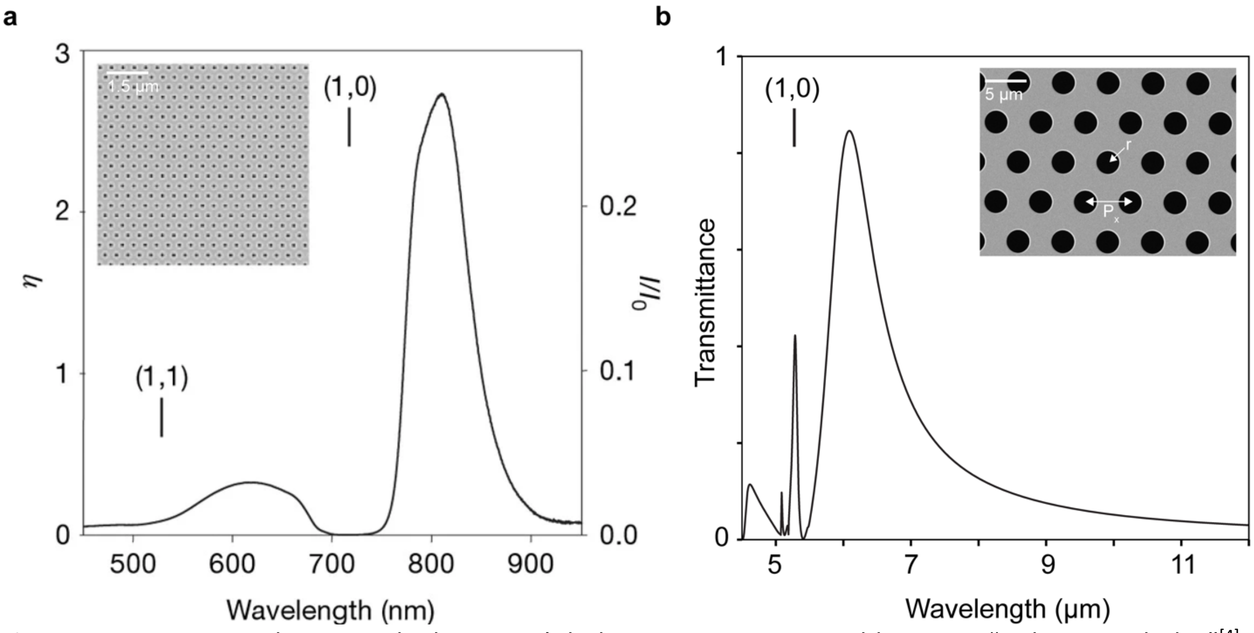

Figure S3. Comparison between the literature's hole array in Genet, C., Ebbesen, T. "Light in tiny holes"[4] and our work.

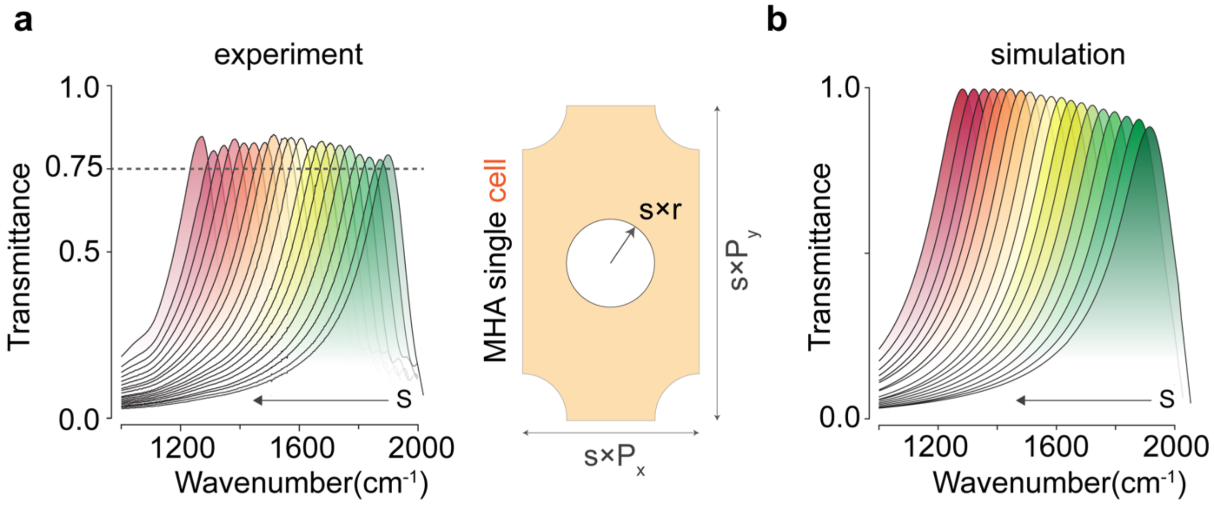

Figure S4. Experimental and simulation MHA spectral response. a, Shows the raw, non-normalized experimental transmittance spectra. In our fabricated metasurfaces, the transmission resonance amplitudes across all resonance modes are larger than 75%. The shape and amplitude of the measured transmittance peaks are in good agreement with the simulation in **b**.

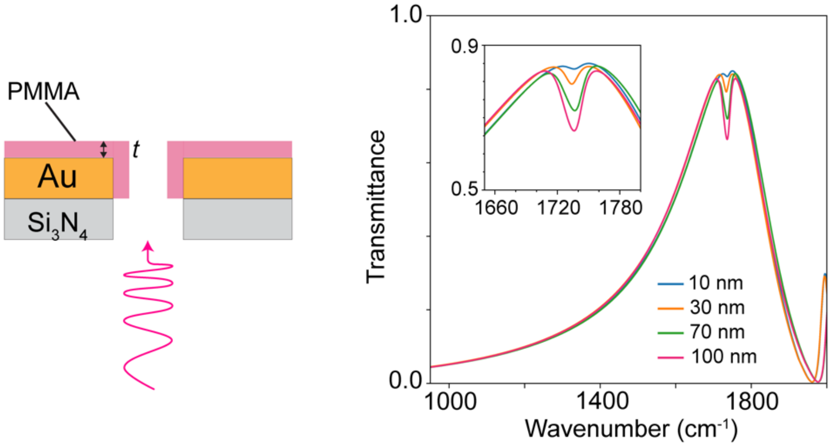

Figure S5. Simulation of the on-resonance MHA response for four distinct PMMA thicknesses (10, 30, 70, and 100 nm). As the PMMA thickness increases, the amplitude of the PMMA band around 1730 cm^{-1} becomes more pronounced (see inset), because a larger number of PMMA molecules interact with the plasmonic mode.

Figure S6 compares the spectra of spin-coated PF on bare CaF_2 versus spin-coated PF on the plasmonic metasurface. On the bare CaF_2 substrate (pink spectrum), the absorption dip in the transmittance at 1656 cm^{-1} corresponding to the most prominent protein band shows only ~4% amplitude. Whereas the same quantity of analyte on the plasmonic metasurface produces a ~40% dip for the identical band at 1656 cm^{-1}.

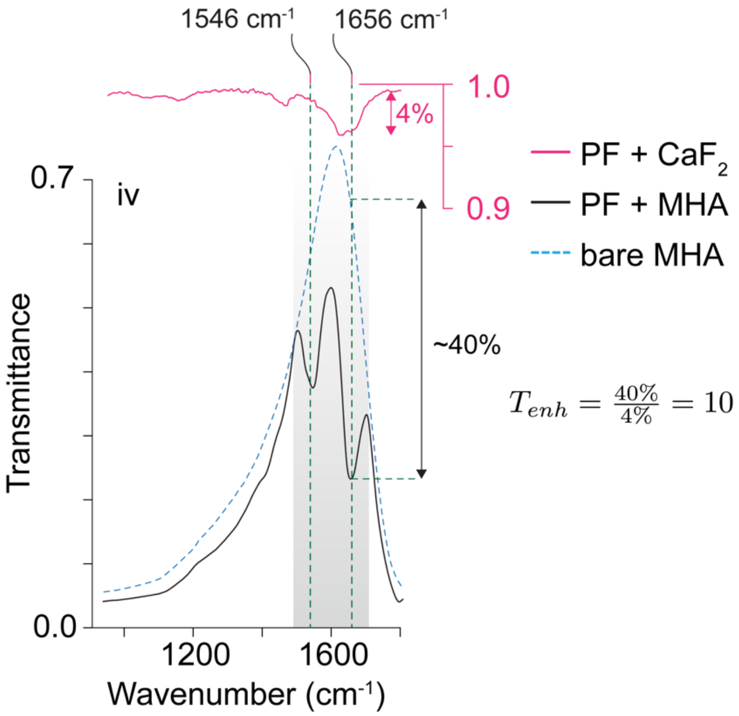

Figure S6. Spin-coated PF on CaF2 vs. Spin-coated PF on the top face of the MHA.

Our plasmonic metasurface enables superior qualitative analysis of fingerprint spectra by providing enhanced spectral discrimination of neighboring bands in congested spectral regions (e.g., protein bands). For example, Figure 6 demonstrates this capability with specific examples: 1) the band at 1590 cm^{-1} captured by MS_{15} shows higher amplitude compared to the spectra from bulk PF samples. 2) the neighboring bands at 1644 cm^{-1} and 1660 cm^{-1} are much better resolved by MS_{16} than in bulk spectra. This improvement stems from a fundamental difference in sampling approach. In transmission spectra from bulk samples, strong bands dominate the signal and mask weaker spectral features. Our SEIRAS approach overcomes this limitation by confining the sampling volume to plasmonic hotspots. This resolves the spectral congestion issues while improving the qualitative analysis of the fingerprint spectra form complex biosamples.

Quantitative analysis of analytes using the plasmonic metasurfaces is achieved by measuring the amplitude of the dips in the resonance transmittance peak of the metasurface and correlating the dip amplitude to the material thickness. For example, Figure S7a, S7b shows simulated spectra of different thicknesses of PMMA layers on the metasurface, revealing that the amplitude of the dip correlates to the thickness of the PMMA layer. Additionally, for quantitative analysis of complex biological samples, we previously used the second derivative spectra[1,2]. In this method, the area above the troughs is measured by integrating the second-order derivative spectra over each band of interest (see Figure S7c). Finally, when our method is combined with powerful data processing or pattern recognition methods, such as ML/AI, both qualitative and quantitative features of the spectra can be smartly captured beyond the capabilities of linear regression approach we show in Figure S7b. We previously demonstrated the use of ML to identify specific collagen molecules from mid-IR spectral features,[3] where a combination of qualitative and quantitative spectral features was used for collagen-dense region segmentation.

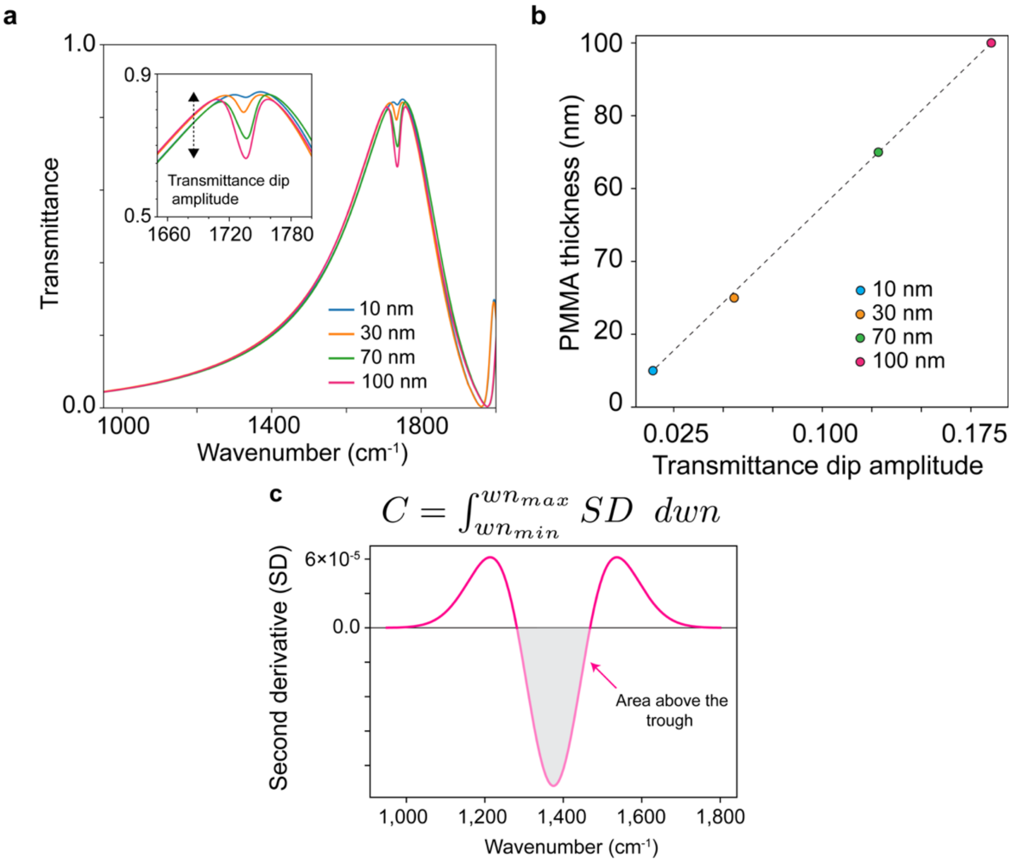

Figure S7. Quantitative analysis methods for SEIRAS applications using MHA. a, Transmittance spectra for varying thicknesses of PMMA on the MHA. **b**, Linear correlation of the transmission dip amplitude with

the PMMA thickness. **c**, An alternative approach for extracting quantitative information from SEIRAS signals is by integrating the area above the troughs of the second-order derivative of the spectra over each band of interest integral.

To benchmark our SEIRAS platform with a typical SEIRAS technology, we fabricated new metasurfaces with hexagonal Au-pillar arrays on a CaF_2 substrate. Specifically for unbiased comparison, we created the inverse design of the MHAs by creating Au pillars instead of holes keeping the geometrical parameters identical. While the MHA array exhibits a peak in transmittance, the Au-pillar array has a resonance dip. To compare their sensing performance, we used the same protocol to deposit a protein monolayer on both metasurfaces and measured them under identical conditions. The Au-pillar metasurface exhibits a broadening and a red shift in resonance dip, without a clear observation of the protein vibrational peak at ~1656 cm^{-1} (see Figure S8a). In contrast, the MHA effectively captures the protein fingerprint in the same spectral region, showing a clear absorbance signal of the protein monolayer (see Figure S8a). These comparative measurements reveal that our MHA metasurface approach is superior to many conventional plasmonic metasurfaces in capturing the SEIRAS signals in transmission mode. This is primarily because of their low transmittance at the resonance, leading to a low number of photons reaching the detector at the valley minimum (T < 10%) close to the detector's noise floor.

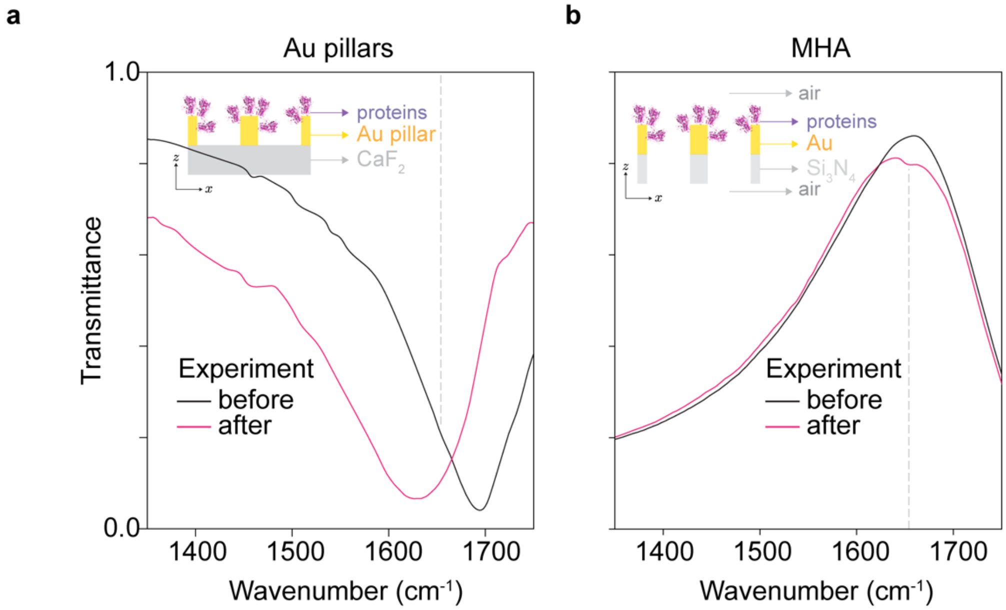

Figure S8. Molecular fingerprint detection of a protein monolayer: performance comparison. a, Au-pillar array before and after deposition of a protein monolayer. Notice that there is no clear signature of the protein band at 1656 cm^{-1}. **b**, The MHA clearly retrieves the vibrational signature of the protein monolayer at 1656 cm^{-1}.

References

[1] S. Rosas, K. A. Schoeller, E. Chang, H. Mei, M. A. Kats, K. W. Eliceiri, X. Zhao, F. Yesilkoy, *Advanced Materials* **2023**, *35*, 2301208.
[2] M. R. Whitbeck, *Applied Spectroscopy* **1981**, *35*, 93.
[3] W. Adi, B. E. R. Perez, Y. Liu, S. Runkle, K. W. Eliceiri, F. Yesilkoy, *JBO* **2024**, *29*, 093511.
[4] C. Genet, T. W. Ebbesen, *Nature* **2007**, *445*, 39.
[5] R. L. Olmon, B. Slovick, T. W. Johnson, D. Shelton, S.-H. Oh, G. D. Boreman, M. B. Raschke, *Phys. Rev. B* **2012**, *86*, 235147.
